# Structural Determinants and the Limits of the Clinical Model in Periodontal Disease

**DOI:** 10.1111/cdoe.70107

**Published:** 2026-08-05

**Authors:** Fábio R. M. Leite, Gustavo G. Nascimento, Marco A. Peres

**Affiliations:** ^1^ School of Dentistry University of Utah Salt Lake City Utah USA; ^2^ Oral Health Academic Clinical Program Duke‐NUS Medical School Singapore Singapore; ^3^ National Dental Research Institute Singapore National Dental Centre Singapore Singapore

**Keywords:** noncommunicable diseases, Oral health policy, periodontal diseases, social determinants of health, universal health coverage

## Abstract

**Background:**

Periodontitis remains one of the most prevalent chronic diseases worldwide and is strongly socially patterned. However, contemporary disease classifications and guideline‐based management largely emphasise individualised, clinically intensive, long‐term maintenance approaches. Whether these models are feasible, equitable, or sustainable when applied at the population level has received little attention.

**Methods:**

We synthesised evidence from global and national epidemiological studies, health systems research, clinical practice guidelines, and health policy analyses to examine the implications of current periodontitis classifications and care models for service demand, workforce capacity, equity, and universal health coverage. We developed a conceptual framework linking disease definitions, recommended care intensity, and health system constraints.

**Results:**

Current periodontitis classifications and maintenance‐oriented care recommendations generate levels of service demand that substantially exceed the capacity of most oral health systems. This creates a feasibility paradox whereby increasingly comprehensive disease definitions and intensive maintenance schedules lead to implicit rationing of care and socially patterned access, disproportionately disadvantaging populations already at highest risk. The prevailing clinical paradigm also places limited emphasis on structural determinants of disease, population prevention, and value‐based allocation of resources.

**Conclusions:**

A population‐oriented reorientation of periodontal care is needed. We propose a framework that prioritises documented disease progression, risk of harm, and person‐centred outcomes; aligns treatment intensity with expected benefit through risk‐based and value‐oriented approaches; and explicitly incorporates health system capacity together with the structural and commercial determinants of health. Moving beyond maintenance‐for‐all models toward feasible, equitable, and system‐aware strategies is essential to reducing the population burden of periodontitis and advancing universal health coverage.

## 1 Introduction

Periodontitis is one of the most common chronic conditions worldwide. Global estimates suggest that severe periodontitis affects over 11% of adults, with prevalence remaining relatively stable over the last three decades and projected to remain broadly stable until 2050. However, the absolute number of people living with the disease has grown in parallel with population growth and ageing [1]. In many settings, periodontitis contributes meaningfully to tooth loss, impaired oral function, and reduced quality of life, particularly in older adults [1, 2]. These burdens are not evenly distributed: They follow clear social gradients, with higher levels of disease and worse outcomes among those who are socioeconomically disadvantaged, marginalised, or exposed to multiple health risks [2, 3, 4]. Yet, despite this substantial and socially patterned burden, the dominant responses to periodontitis continue to focus primarily on individualised clinical care.

The dominant approach to periodontal disease prevention and management remains anchored in a narrow clinical and behaviour‐focused model. In this model, dental biofilm is framed as the primary cause of disease, patient behaviour as the main modifiable determinant, and scaling, root planing, and frequent maintenance visits as the central (and in many cases “only”) tools for disease prevention and control [5, 6]. The widely promoted expectation is that if applied intensively enough, this configuration of care will reduce the incidence and burden of periodontitis at the population level.

We contend that this expectation has long been questionable [7]. First, periodontitis is shaped by a constellation of structural and commercial determinants—including poverty, precarious work, limited access to health care, tobacco use, unhealthy diets, psychosocial stress and broader social inequalities—that cannot be adequately addressed by chairside interventions alone [3, 8, 9, 10, 11, 12, 13]. Second, the way periodontal disease is currently classified and clinical guidelines are formulated tends to expand the category of “disease” and prescribe follow‐up regimens that are unfeasible at scale, even in well‐resourced systems [5, 6, 14, 15, 16]. This creates what we refer to as a feasibility paradox: The more comprehensively we define and seek to treat “periodontitis” at the individual level, the less realistic it becomes to offer equitable care.

Brazil provides a useful example of these tensions, not as an outlier but as an illustrative case of dynamics observable across diverse health systems. As we detail below, its experience shows how improvements in clinical provision, although valuable, have limited capacity to alter the underlying incidence of disease when structural conditions remain largely unchanged. Similar patterns in countries with quite different health systems suggest the problem lies less in the diligence of clinicians than in the conceptual and policy frameworks in which they operate [2].

Our aim is not to dismiss the advances in clinical periodontology, nor to deny the effectiveness of non‐surgical therapy, surgical treatment, and supportive care for individual patients under favourable conditions. Our argument is that, when these clinical successes are extrapolated uncritically to the population level and treated as the primary levers for reducing incidence and inequalities, they generate unrealistic expectations and divert attention from structural and system‐level change.

This paper examines the population‐level implications of applying contemporary periodontal disease classifications and guideline‐based care models at scale, questioning the consequences of translating these frameworks from the individual patient to health systems and populations. We show that widely accepted assumptions underpinning current clinical practice, regarding disease definition, behavioural control, and maintenance‐based care, generate levels of service need that are incompatible with workforce capacity, financing, and equity goals in most—if not all—health systems (Figure 1). Our contribution lies in reframing periodontal disease through a health systems and structural determinants lens, highlighting pathways toward more feasible, equitable and value‐oriented models of care. Table 1 summarises the key differences between the traditional clinical model and the population‐, risk‐ and structure‐oriented approach proposed here, across conceptual, strategic, and health system dimensions.

**FIGURE 1:**
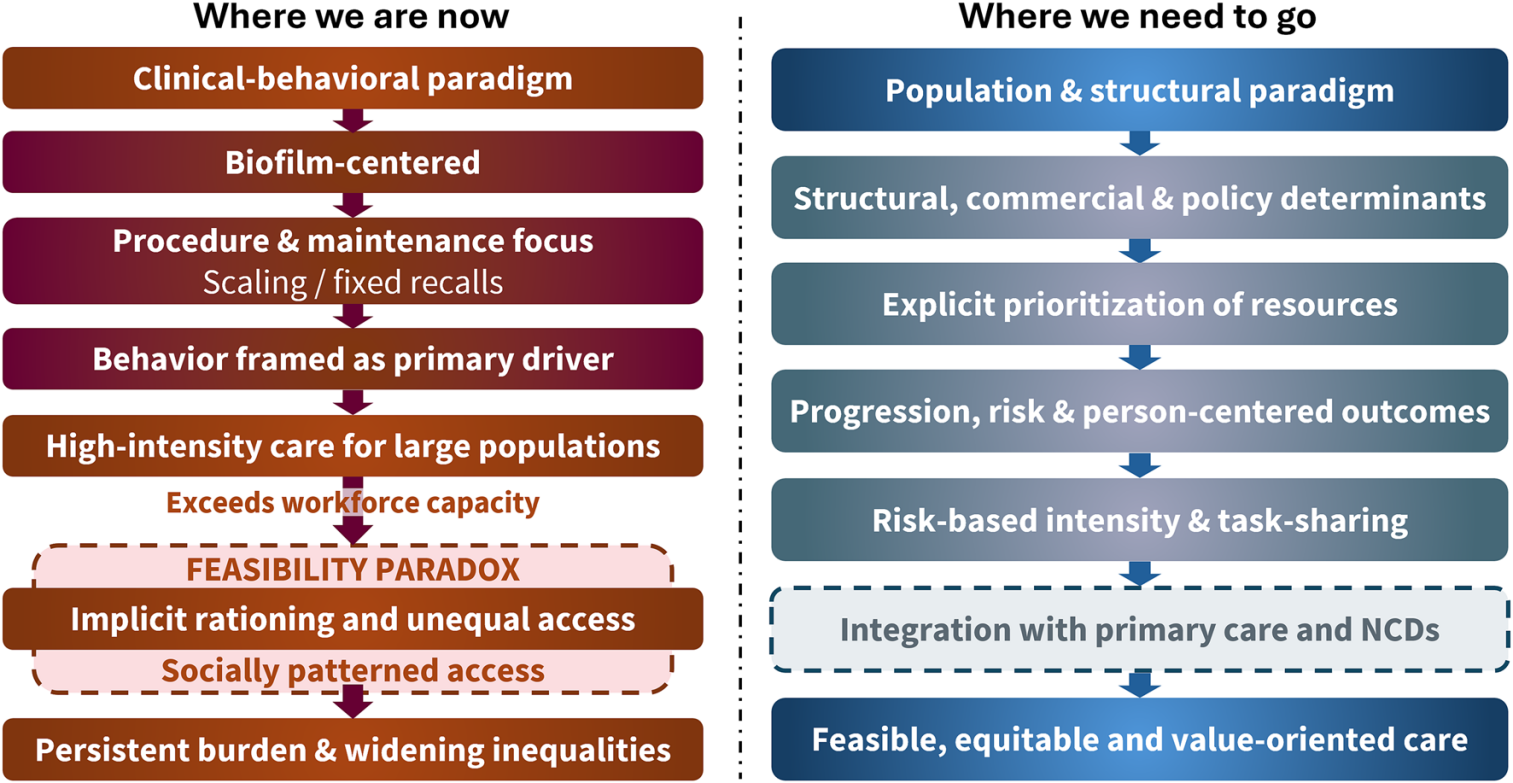
Schematic representation of the transition from a traditional clinical‐behavioural model to a population‐, risk‐ and structure‐oriented approach to periodontal care. The left panel depicts the current dominant pathway, in which a clinical‐behavioural paradigm centred on biofilm, procedural maintenance, individual behaviour, and uncertainty surrounding disease progression drives high‐intensity care for large populations, exceeds workforce capacity, and produces a feasibility paradox characterised by implicit rationing, socially patterned access, and persistent burden and widening inequalities. The right panel depicts the proposed reorientation, grounded in structural, commercial, and policy determinants, explicit resource prioritisation, progression‐ and risk‐based disease definitions, person‐centred outcomes, risk‐based care intensity and task‐sharing, and integration with primary care and non‐communicable disease strategies, resulting in feasible, equitable and value‐oriented care. NCD, Non‐Communicable Disease.

**TABLE 1:** Contrasting frameworks for periodontal care: From a traditional clinical model to a population‐, risk‐ and structure‐oriented approach.

Aspect	Traditional Clinical Model	Population‐, Risk‐ and Structure‐Oriented Model
Concept of disease	Cross‐sectional presence of attachment loss or pockets	Documented progression, risk of clinically meaningful harm, person‐centred impact
Primary focus	Biofilm and individual behaviour	Disease progression, risk stratification, functional and quality‐of‐life outcomes
Implicit theory of causation	Individual biology and behaviour aggregated to populations	Structurally patterned risk shaped by social, commercial and policy environments
Care strategy	Routine, fixed‐interval maintenance for large population groups	Intensity and frequency tailored to risk, benefit, and system capacity
Interventions and delivery strategies	Scaling, root planing, intensive oral hygiene instruction	Risk‐based screening, basic non‐surgical care, task‐sharing, integration with NCD care
Role of guidelines	Prescriptive, procedure‐ and maintenance‐oriented	Flexible, prioritisation‐oriented, evidence‐based thresholds for treatment need aligned with feasibility and equity
Scalability and feasibility	Low – generates workforce and resource demands that exceed system capacity	Higher – explicit prioritisation and resource allocation to those most likely to benefit
System response to scarcity	Implicit rationing and unequal access	Explicit prioritisation and transparent allocation
Consideration of social and commercial determinants	Peripheral or implicit	Central – tobacco, diet, work, living conditions, commercial influences
Equity impact	Tends to reinforce inequalities through unequal distribution of resources	Oriented toward reducing inequities in access and outcomes
Integration with general health	Limited	High – aligned with primary care, UHC and NCD strategies

Abbreviations: NCD, Non‐Communicable Disease; UHC, Universal Health Coverage.

## 2 Where Are We Now? Structural and Systemic Limits of the Prevailing Model

### 2.1 The Persistent Global Burden of Periodontitis

As outlined in the Introduction, the epidemiology of periodontitis over recent decades combines persistence with expansion. Although age‐standardised prevalence has remained relatively stable, population growth and ageing have increased the absolute number of people classified as experiencing periodontal destruction and its consequences across the life course [1]. At the same time, these estimates are inherently shaped by the case definitions and classification systems used to identify disease, which commonly rely on simplified and partly arbitrary clinical thresholds such as probing pocket depth and clinical attachment loss. Such choices may overestimate prevalence and obscure variation in disease extent, progression, and functional impact. Nevertheless, in the absence of widely accepted alternatives for population‐level surveillance and longitudinal monitoring, these clinical parameters remain necessary, albeit imperfect, tools for describing disease patterns over time. Within this context, periodontal destruction should be understood as existing along a continuum of severity, progression, and functional impact, rather than as a simple dichotomy of “disease” vs. “no disease.” Consequently, even where prevalence curves appear relatively flat, the overall burden attributed to periodontitis—including impaired oral function, impacts on tooth retention and quality of life, and years lived with disability—remains substantial and socially patterned [1]. A thorough discussion on the classification of periodontitis is beyond the scope of this article [16].

At the country level, this broad pattern is repeated. In many high‐income settings, adults are now retaining more teeth into older age, increasing the proportion of the population potentially exposed to periodontal breakdown over the life course [1]. However, greater tooth retention and longer exposure to periodontal risk do not necessarily translate into proportional increases in clinically significant or patient‐important disease outcomes, as improvements in periodontal health indicators have also been observed in some populations despite increased tooth retention [17]. In lower‐ and middle‐income countries, epidemiological transition and partial expansion of dental care have occurred against a backdrop of persistent social and regional inequalities [2]. Although clinical services may be more available than in the past, substantial disparities remain in access to timely and effective care, and patterns of periodontal burden continue to reflect socially patterned differences in risk exposure, care access, and historical unmet need across the life course [1, 2].

Importantly, these aggregate indicators conceal systematic differences in who develops disease and who experiences its consequences. Within virtually all countries where data are available, periodontitis and severe tooth loss are more frequent among people with lower income and education, those in precarious employment, and those living in deprived neighbourhoods or rural areas [1, 18]. These gradients are mirrored in comorbidities such as diabetes, cardiovascular disease, and obesity, pointing to shared upstream determinants rather than isolated, individual‐ and tooth‐level risk factors [4, 7, 12, 19, 20, 21].

### 2.2 Brazil as an Illustrative Case

Over recent decades, the country has implemented substantial reforms in oral health, including the expansion of primary dental care teams within the Unified Health System (Sistema Único de Saúde, in Portuguese), the integration of oral health into broader primary care strategies, as well as the growth of secondary (specialised) and tertiary oral care, contributing to increased coverage of dental services and improvements in some indicators of oral health, such as reduced prevalence of untreated caries in children [2, 22, 23].

Yet, when one examines periodontal outcomes among adults and older adults, a more complex picture emerges. National epidemiological surveys have consistently documented a high prevalence of periodontal pockets and clinical attachment loss, alongside substantial tooth loss in later life [1, 22, 23]. Older Brazilians frequently present with a history of extractions and partial edentulism rather than successful long‐term periodontal maintenance. Smoking, a major causal risk factor for periodontitis, remains socially patterned in Brazil and interacts with broader structural and commercial determinants of health, contributing to unequal distributions of periodontal disease and tooth loss across populations [24]. These patterns vary across regions and social groups, with a higher burden observed in poorer regions, among individuals with lower schooling, and among those without stable access to care [3, 22, 23]. Recent analyses of national survey data further suggest that, although some indicators of periodontal health have improved over time, these gains have been unevenly distributed, with persistent socioeconomic inequalities in periodontal outcomes across the Brazilian population [25].

This configuration reflects the interaction of several factors. Many adults enter the system only when symptoms become severe enough to interfere with daily life. Preventive and supportive periodontal care is often intermittent, constrained by service organisation, workforce distribution, and competing demands within primary care [22, 23]. At the same time, structural conditions, including income inequality, food environments, working conditions, and exposure to commercial determinants such as tobacco and ultra‐processed foods, continue to fuel the incidence and progression of disease [3, 4, 8, 9, 12, 20, 21, 26, 27]. The result is a scenario in which expansions in clinical provision, while necessary and beneficial, have only modest capacity to transform the overall pattern of periodontal burden and inequalities.

Although specific features differ, similar tensions can be observed in countries with very different health systems and income levels [1]. This suggests that the problem is not unique to Brazil, but emblematic of a broader disconnect between the epidemiology of periodontitis and the way periodontal care has been conceptualised and organised globally.

### 2.3 False Certainties in the Clinical Narrative

Taken together, these patterns reflect a cluster of assumptions that operate as “false certainties” within dominant clinical reasoning. These certainties appear logical within the clinic but obscure the broader social and structural forces shaping disease, as illustrated in Figure 1. They can be grouped into three domains: How disease is understood, how behaviour is interpreted, and how guidelines are presumed to function at scale.

False certainty 1 is the assumption that understanding periodontitis at the level of the site, the tooth, the biofilm, and the individual is sufficient to explain and alter its distribution at the population level [14, 28]. Within this framework, population patterns of periodontitis are implicitly treated as the aggregate result of individual biological susceptibility and behavioural choices, modifiable primarily through chairside clinical interventions and patient education. Current periodontal practice is also supported by a second, related assumption arising from uncertainty surrounding disease progression: Because clinicians cannot reliably predict which individuals will experience clinically significant progression, less selective and more intensive treatment strategies are often adopted as a precautionary response to uncertainty. Together, these perspectives reinforce high‐intensity, population‐wide approaches to care centred on individualised intervention and maintenance. This orientation is reflected in contemporary classification systems and consensus reports, which prioritise site‐specific clinical parameters and biofilm‐mediated mechanisms as central explanatory pathways for disease occurrence and progression [5, 6], as well as in supportive periodontal therapy literature that emphasises patient adherence and “compliance” as key determinants of long‐term outcomes [29].

This certainty obscures the fact that population distributions of periodontitis are shaped by structural forces that operate upstream of individual behaviour, including income inequality, educational opportunities, labour conditions, housing, and access to health care. These forces pattern both exposure to risk factors and the capacity to benefit from clinical care, producing social gradients that cannot be eliminated through individualised treatment alone [30] and reflect lifecourse differences in risk, access, and prior unmet need, rather than an inevitable accumulation of disease with age [2, 7, 18, 19]. At a systems level, these false certainties lead to the misalignment of explanatory models and policy responses, privileging clinical and behavioural interventions while underinvesting in structural policies capable of shifting the population distribution of disease [31].

False certainty 2 is the belief that individual‐level behavioural counselling—focused on oral hygiene practices, smoking cessation, and lifestyle modification—can effectively offset the influence of powerful structural and commercial determinants of periodontitis. Within this narrative, failure to achieve periodontal stability is frequently attributed to inadequate adherence or “non‐compliance” by patients (“victim‐blaming approach”), rather than to the environments in which behaviours are produced and constrained [21, 32]. This framing underestimates the role of commercial practices and social conditions that systematically shape exposure to tobacco, unhealthy diets, psychosocial stress and precarious living and working environments. Behavioural choices are not made in a vacuum; they are socially patterned and strongly conditioned by policies, markets, and material circumstances [19, 30]. Within this framework, preventive care is also frequently conceptualised primarily in terms of individualised chair‐side interventions and behavioural counselling, often under the assumption that “prevention is better than cure” irrespective of the magnitude or sustainability of their effects at the population level. While preventive strategies targeting structural and commercial determinants, such as tobacco control, fiscal policies, and broader public health measures, have demonstrated substantial population benefits, the effectiveness of individualised preventive interventions alone is more variable and context‐dependent [33]. In practice, this false certainty legitimises a moralised view of risk, in which responsibility for disease control is shifted onto individuals, while the upstream drivers of periodontitis remain largely unchallenged. The result is an intervention mix that places high demands on patients least able to meet them, thereby reproducing social inequalities in periodontal outcomes [14, 28, 34].

False certainty 3 is the assumption that contemporary periodontal classifications and clinical practice guidelines—developed largely in well‐resourced settings—can be implemented at scale in a feasible and equitable manner. Importantly, the tensions described here are not unique to contemporary frameworks. Earlier periodontal indices and classifications, including the Community Periodontal Index (CPI), were also criticised for imprecision and for potentially overestimating treatment needs when applied at population level [35]. Broad case definitions combined with recommendations for intensive, often indefinite, maintenance create an implicit expectation that health systems can deliver frequent professional care to all individuals classified as having periodontal diseases [5, 6]. While earlier approaches were often criticised for being overly simplistic or insufficiently sensitive to disease heterogeneity, more recent classifications—although more refined and biologically detailed—may similarly expand the population defined as requiring ongoing care when translated into guideline‐based practice. When these classifications are translated into population‐level estimates of service need, a feasibility paradox emerges. Even conservative assumptions regarding visit duration, recall intervals and workforce availability suggest that delivering guideline‐concordant care to all eligible individuals would require levels of time, personnel and financial resources that exceed what is available in most health systems, including many high‐income countries [14, 28, 34, 36]. Because this infeasibility is rarely acknowledged explicitly, health systems respond through implicit rationing, in which access to intensive periodontal care is uneven and socially patterned. Those with greater resources and social capital are more likely to receive sustained maintenance, while disadvantaged groups experience episodic, delayed, or absent care, thereby reinforcing the very inequalities that clinical guidelines purport to address [14, 28, 34, 36].

## 3 Reframing What We Call “Disease” and How We Define Success

If current classifications and guidelines risk generating unfeasible levels of “need” and unrealistic expectations of clinical workload, one starting point for change is to reconsider how we define periodontal disease and what we regard as successful care [5, 6, 14, 15, 16]. Rather than equating relatively small amounts of attachment loss or shallow pockets with “disease” requiring intensive, indefinite maintenance, we argue for a stronger emphasis on three elements: Documented progression over time, risk of clinically meaningful harm, and person‐centred outcomes [14, 28, 34, 36]. Disease definitions serve multiple functions beyond guiding treatment—communication, resource allocation, and explanation of presenting problems—so the aim is to rebalance, not replace, the criteria that drive care intensity, while preserving the communicative and administrative roles these definitions must continue to serve [16, 37]. Periodontitis is not unique in facing these tensions. The expansion of disease definitions, and the resulting risks of overdiagnosis and overtreatment, has been examined across medicine, including in philosophical analyses of how the boundary between health and disease is drawn and in calls to reframe conditions in terms of lived experience and suffering rather than clinical thresholds alone [38, 39]. A recurring lesson from these wider debates is procedural: Revisions to disease definitions and guidelines are more trustworthy when developed through transparent processes that explicitly weigh the benefits and harms of broadening a definition and incorporate the values and preferences of patients and the wider community, rather than relying on expert consensus alone [16, 40]. Frameworks such as the Checklist for Modifying Disease Definitions offer one structured route to this, and periodontology would benefit from adopting similarly explicit and inclusive approaches when revising case definitions and care guidelines [16]. These perspectives reinforce our argument that how periodontitis is defined, and not only how it is treated, has direct consequences for feasibility, equity, and the goals of care.

First, progression matters. A single cross‐sectional examination cannot distinguish between historical, now‐stabilised destruction and active disease. In resource‐constrained systems, it is neither necessary nor realistic to treat every trace of historical tissue loss with the same intensity as rapidly progressing disease [14, 28]. Contemporary periodontal classifications already incorporate concepts related to progression and future risk, including grading and tooth loss attributable to periodontitis. However, determining whether tissue destruction reflects ongoing activity, historical damage, or meaningful future risk remains challenging in routine clinical practice, particularly when decisions rely heavily on cross‐sectional findings. Greater emphasis on documented change over time and the likelihood of clinically meaningful deterioration may help better calibrate care intensity to the actual distribution of harm within populations [34, 41]. This includes prioritising individuals at greater risk of tooth loss, functional impairment, or progressive destruction, while avoiding unnecessarily intensive long‐term management for individuals with stable conditions and low risk of future harm.

Second, clinical relevance and risk of harm should be central. Periodontal conditions vary substantially in their symptoms, functional consequences, impact on quality of life, and likelihood of benefiting from intensive long‐term management. Yet, in practice, broad diagnostic categories may still channel diverse patient groups toward similar maintenance‐oriented care pathways. Not all attachment loss translates into functional impairment, pain, or a substantial risk of future tooth loss [42, 43]. In this context, treatment thresholds and maintenance recommendations should be calibrated not only to the presence of clinical signs but also to the expected magnitude of benefit, patient priorities, and the broader opportunity costs associated with intensive long‐term care [14, 44, 45]. Such an approach may help prioritise resources toward individuals most likely to benefit from more intensive intervention while avoiding unnecessary treatment burden and dilution of limited system capacity across large groups with relatively limited clinical impact.

Third, the goals and success of periodontal care need to be defined in ways that are meaningful to patients and responsive to clinical, social, and system contexts. This includes the preservation of comfortable, functional dentition; the ability to eat, speak, and socialise without embarrassment; and the avoidance of preventable tooth loss [34, 46, 47]. Importantly, successful care should not be understood as a fixed or exclusively clinician‐defined endpoint based solely on achieving specific clinical parameters. Rather, it may also include informed, autonomous patient decisions to defer or decline treatment, particularly where expected benefits are uncertain, burdens are substantial, or patient preferences and priorities differ from professional norms. Person‐centred outcomes can therefore be used alongside traditional clinical indicators to guide both individual care and service evaluation. Different treatment goals and care strategies should also remain open to empirical evaluation, including through pragmatic and mixed‐methods research assessing their effects on patient‐important outcomes, costs, treatment burden, and health system feasibility. Reframing disease and success in this way does not mean lowering standards; it means aligning our metrics with realistic, meaningful goals at both clinical and population levels.

## 4 From Maintenance‐For‐All to Risk‐Based, Value‐Oriented Care

A further area for change concerns the organisation of care. The current tendency to recommend short, fixed‐interval maintenance for large proportions of the adult population is difficult to reconcile with finite resources and large care gaps [14, 48]. Notably, the evidence underpinning routine fixed‐interval recall is itself limited; trial data provide little support for universally applied short‐interval scaling and polishing in terms of patient‐important outcomes, which makes the resource demands of maintenance‐for‐all still harder to justify [33]. Risk‐based and value‐oriented models of care offer a more defensible alternative.

We acknowledge that some guidelines and health systems have begun to incorporate elements of risk‐based recall and multidisciplinary management, and that individual clinicians often adapt recommendations pragmatically to local constraints. However, these developments remain uneven and are frequently layered onto payment and organisational structures that continue to reward volume over value. As a result, risk‐based rhetoric often coexists with practice patterns that still assume short‐interval maintenance for large proportions of the adult population, without explicit prioritisation of those at greatest risk or need.

Risk‐based models tailor the intensity and frequency of supportive periodontal therapy to the individual's likelihood of progression and harm, taking into account systemic conditions, smoking status, prior history of rapid progression, and social factors that may shape exposure and adherence [48, 49]. Patients at low risk of progression, with stable periodontal status and good self‐care, may be safely managed with less frequent visits, whereas those at higher risk may appropriately receive more intensive follow‐up and multidimensional support [50, 51, 52].

Value‐oriented care—understood here as care that prioritises outcomes meaningful to patients [34], while using finite system resources to deliver those outcomes equitably across the population rather than maximising the volume of procedures [53]—requires that financing and organisation reward those outcomes rather than activity alone [54]. In many systems, current payment structures incentivise repeated instrumentation and high‐frequency recall without necessarily ensuring that those most in need receive appropriate care [34, 53]. Seen in this light, the maintenance‐for‐all model is not a neutral clinical default but is itself shaped by professional, organisational, and commercial incentives that can favour high‐intensity, procedure‐based care independently of demonstrated population benefit—sustaining demand for services while attributing shortfalls to individual patients rather than to the model itself.

From a health system perspective, this is also a question of opportunity cost: Resources devoted to intensive maintenance for relatively low‐risk individuals cannot simultaneously be used to expand access to basic periodontal and restorative care for underserved populations or to invest in upstream preventive policies. Reorienting toward value could include linking reimbursement to indicators of stability and tooth retention, integrating periodontal care into broader chronic disease management packages, and supporting models of care that expand access to preventive and supportive services in primary care settings [53, 54]. By reallocating routine preventive and maintenance tasks to appropriately trained non‐dentist providers, such models can also reduce the cost per unit of periodontal health gained and free up specialist capacity for more complex care [55].

A shift toward risk‐based and value‐oriented care also has implications for the oral health workforce (Figure 1). In most health systems, dentists are unevenly distributed and concentrated in better‐resourced areas [56], which makes it unrealistic to rely solely on specialist or dentist‐delivered care to implement periodontal prevention and maintenance at scale. Strengthening and appropriately regulating the roles of dental hygienists, dental therapists, oral health technicians [55], and community health workers can support the delivery of risk‐based periodontal care in primary care settings, including screening, basic non‐surgical therapy, reinforcement of self‐care and linkage with broader non‐communicable diseases (NCD) management. This team‐based approach can foster closer collaboration and trust (“bonding”) between dentists and other oral health professionals, with dentists focusing their time on diagnosis, complex non‐surgical and surgical management, integration with medical care for people with multimorbidity, and clinical leadership and supervision [55]. Investing in such team‐based care models, and in the training and retention of these cadres, is therefore essential if the strategies we propose are to be implemented equitably rather than only in well‐served population groups [18, 57].

Brazil's experience again provides a useful example. The expansion of public oral health teams within the Unified Health System—typically composed of a dentist, oral health technicians or assistants and, in many municipalities, community health workers linked to the Family Health Strategy—has created opportunities to integrate periodontal care into primary care, but capacity remains limited, and demand is high [23, 57]. In principle, these multiprofessional teams can share tasks across cadres, with non‐dentist oral health professionals playing central roles in population outreach, screening, basic non‐surgical therapy, reinforcement of self‐care and linkage with broader NCD management [55]. In practice, however, scope‐of‐practice rules, training gaps and workload pressures often mean that periodontal prevention and maintenance remain heavily concentrated in dentist‐delivered care. Implementing simplified risk‐stratification tools, expanding and supporting the roles of these other oral health professionals, focusing specialist resources on complex or rapidly progressing cases, and linking clinical decision‐making to explicit goals of tooth retention and function could help to use available resources more equitably and effectively [34, 57]. Similar principles can be observed in high‐income countries, where large numbers of adults are assigned intensive maintenance schedules that may offer diminishing marginal returns relative to their cost [14, 28, 36].

## 5 Putting Structural and Commercial Determinants at the Centre of Periodontal Health Policy

Perhaps the most fundamental shift required is conceptual: Periodontal disease needs to be understood and addressed as a structurally determined, commercially influenced condition, not solely as a problem of biofilm, individual behaviour, and chairside care [2, 7, 19, 26]. At the population level, some of the most powerful levers for reducing periodontal incidence and progression lie in policies traditionally associated with non‐communicable disease prevention more broadly. Foremost among these is comprehensive tobacco control, the determinant for which the causal evidence in periodontitis is strongest and most consistent [8, 9, 58, 59]. Fiscal and regulatory measures to reduce consumption of free sugars and ultra‐processed foods, policies to improve working and living conditions, and investments in social protection, education, and primary health care are biologically plausible and increasingly studied, although the evidence linking them specifically to periodontitis is still emerging and not yet consistent [13, 19, 26]. Such measures alter the upstream distribution of risks that drive both periodontitis and its comorbidities, including diabetes and cardiovascular disease [3, 4, 20, 27].

From this vantage point, Brazil's relatively strong history of tobacco control, with large reductions in smoking prevalence over recent decades, can be seen as a major periodontal intervention, even if it was not framed as such [3, 58, 59]. Conversely, the rapid expansion of ultra‐processed food markets in many low‐ and middle‐income countries represents an emerging threat to periodontal health via diet‐related metabolic and inflammatory pathways [3, 26, 60, 61, 62]. Recognising these links can help dental professionals and organisations participate more actively in debates on fiscal, trade, and marketing policies that shape oral and general health [2, 19].

Repositioning structural and commercial determinants at the centre of periodontal policy also requires changes in research and advocacy. Research agendas need to move beyond individual‐level risk factor models to investigate how policies, institutions, and corporate practices shape the distribution of periodontal disease and access to care [18, 26, 63]. Professional organisations can use their influence to support policies that reduce exposure to harmful commodities, promote healthy environments, and protect public health regulation from undue commercial interference [2, 18, 57].

## 6 Aligning Periodontal Therapy With Universal Health Coverage and Integrated Non‐Communicable Disease (NCD) Strategies

Finally, if periodontitis is to be addressed as part of a broader equity and universal health coverage agenda, periodontal care must be more fully integrated into primary care and NCD strategies [2, 18, 19, 64]. The current separation of oral health from general health policy, financing and service delivery undermines the ability of systems to respond to shared determinants and multimorbidity.

Integration can take multiple forms. At the clinical level, this includes embedding basic periodontal assessment and counselling into chronic disease management pathways in primary care [65], ensuring that individuals with diabetes, cardiovascular disease and other NCDs receive opportunistic periodontal screening and appropriate referral. It also involves training non‐dental health professionals to recognise signs of periodontal problems and to deliver consistent messages about tobacco, diet, and oral hygiene [2, 66]. Because the effectiveness and cost‐effectiveness of such integration and screening pathways are not yet firmly established, these models should be implemented alongside evaluation rather than assumed to deliver benefit. Moreover, because dental attendance is already widely recommended, the marginal value of additional screening pathways depends largely on whether they reach people not already served by existing care—disproportionately those facing the greatest social and access barriers. The priority may therefore lie less in adding new layers of complexity than in ensuring that recommended visits actually occur and that identified needs are followed, particularly among underserved groups. Where integration is pursued, simpler approaches focused on access and follow‐up warrant evaluation before more elaborate models are adopted.

At the policy and financing level, integration implies including essential periodontal services in universal health coverage benefit packages, at least for those at higher risk and those already living with disease [57]. This requires explicit decisions about which services are prioritised, at what level of care, and with what degree of financial protection [67, 68]. While contexts differ, a common principle is that lack of ability to pay should not be a barrier to receiving at least a basic package of effective periodontal care, particularly for conditions that intersect so directly with other chronic diseases [67, 68].

Internationally, incorporating periodontal indicators into monitoring frameworks for NCDs and universal health coverage could help to make periodontal health more visible to decision‐makers [2, 57]. This would also encourage countries to collect better data on periodontal status, distribution, and care coverage, moving beyond small, unrepresentative clinical samples toward robust surveillance systems that can inform policy and track progress [1, 57].

## 7 Conclusion

Periodontitis exemplifies the limitations of a narrow, clinical, and behaviour‐focused configuration of care in addressing structurally patterned disease. Despite decades of advances in understanding biofilm, host response, and individual risk factors, the global burden of periodontitis remains high, and social gradients in disease and tooth loss persist. As long as our classificatory frameworks, guidelines and service models are organised around intensive, procedure‐ and maintenance‐focused care for large proportions of the population, the gap between what is recommended and what can be delivered equitably will continue to widen.

We have argued that a reorientation is necessary along three main lines. First, periodontal disease definitions and success criteria should place greater emphasis on progression, risk of clinically meaningful harm, and person‐centred outcomes, rather than treating all historical tissue loss as equivalent and indefinitely actionable. Second, care should be organised on a risk‐based and value‐oriented basis, so that the intensity of supportive therapy reflects both need and potential benefit, and financing mechanisms reward stability, tooth retention, and improved function rather than volume of procedures. Third, periodontal policy must move beyond the dental chair to engage with structural and commercial determinants, and to integrate periodontal care within broader universal health coverage and non‐communicable disease strategies.

In practical terms, this implies at least four priority actions for the field: (1) piloting and evaluating progression‐ and outcome‐based case definitions in real‐world settings; (2) testing risk‐based, value‐oriented care pathways within primary care and specialist services; (3) embedding periodontal considerations into national NCD and universal health coverage strategies; and (4) strengthening research and advocacy on the structural and commercial determinants of periodontal health. These steps would help translate the conceptual shift we propose into implementable change.

These shifts will not be straightforward. They require changes in clinical culture, research priorities, professional education, and policymaking. However, they are consistent with wider movements in public health and health services toward equity, person‐centredness, and system feasibility. Periodontology has reached a crossroads similar to those previously encountered in cardiovascular epidemiology, diabetes prevention, and tobacco control. Continuing to rely predominantly on individualised treatment and lifelong maintenance care, while necessary for many patients, is unlikely to substantially reduce the population burden of periodontitis or narrow persistent social inequalities in oral health. Future progress will depend on complementing high‐quality clinical care with policies that address structural and commercial determinants, align care with health‐system feasibility, and prioritise equity. Only through this broader reorientation can periodontology move toward a model of care that is biologically sound, socially just, and sustainable at the population level.

## Author Contributions

F.R.M.L. contributed to study conception and design, data analysis and interpretation, manuscript drafting, and critically revised the manuscript. G.G.N. contributed to study conception and design, data analysis and interpretation, and critically revised the manuscript. M.A.P. contributed to study conception and design, data analysis and interpretation, and critically revised the manuscript.

## Funding

The authors have nothing to report.

## Conflicts of Interest

The authors declare no conflicts of interest.

## Data Availability

This article is based on published literature and does not report any original datasets generated or analysed by the authors. Therefore, no data is available, and data sharing is not applicable.
